# Design and Biological Evaluation of Antifouling Dihydrostilbene Oxime Hybrids

**DOI:** 10.1007/s10126-018-9802-z

**Published:** 2018-03-13

**Authors:** Lindon W. K. Moodie, Gunnar Cervin, Rozenn Trepos, Christophe Labriere, Claire Hellio, Henrik Pavia, Johan Svenson

**Affiliations:** 1 Department of Chemistry, UiT The Arctic University of Norway, Breivika, N-9037 Tromsø, Norway; 2 Present Address: Department of Chemistry, Umeå University, SE-901 87 Umeå, Sweden; 3 Department of Marine Sciences – Tjärnö, University of Gothenburg, SE-452 96 Strömstad, Sweden; 4 Université de Bretagne Occidentale, Biodimar/LEMAR UMR 6539, Rue Dumont d’Urville, 29280 Plouzané, France; 5 Department of Chemistry, Material and Surfaces, RISE Research Institutes of Sweden, Box 857, SE-501 15 Borås, Sweden

**Keywords:** Antifouling, Dihydrostilbene, Batatasin, Oxime, Ianthelline, Hybrid

## Abstract

By combining the recently reported repelling natural dihydrostilbene scaffold with an oxime moiety found in many marine antifoulants, a library of nine antifouling hybrid compounds was developed and biologically evaluated. The prepared compounds were shown to display a low antifouling effect against marine bacteria but a high potency against the attachment and growth of microalgae down to MIC values of 0.01 μg/mL for the most potent hybrid. The mode of action can be characterized as repelling via a reversible non-toxic biostatic mechanism. Barnacle cyprid larval settlement was also inhibited at low μg/mL concentrations with low levels or no toxicity observed. Several of the prepared compounds performed better than many reported antifouling marine natural products. While several of the prepared compounds are highly active as antifoulants, no apparent synergy is observed by incorporating the oxime functionality into the dihydrostilbene scaffold. This observation is discussed in light of recently reported literature data on related marine natural antifoulants and antifouling hybrids as a potentially general strategy for generation of improved antifoulants.

**Electronic supplementary material:**

The online version of this article (10.1007/s10126-018-9802-z) contains supplementary material, which is available to authorized users.

## Introduction

Marine biofouling, the rapid colonization and growth of organisms on marine surfaces, commonly occurs on ships, buoys, cooling systems, and aquaculture equipment (Yebra et al. 2004). The biofouling film forms rapidly on submerged substrata and often contains both marine microorganism and macroorganism. The biofilm often requires removal on these industrial structures, and the costs associated with maintenance, infrastructure damage, and increased fuel costs due to reducing shipping efficiency are significant (Callow and Callow 2011; Schultz et al. 2011). Surface treatment with paint-containing biocidal compounds proved an effective strategy to counter biofouling for many decades; however, this was accompanied with adverse effects to the surrounding marine environments (Alzieu et al. 1986). For example, the once commonly used tributyl tin is both persistent and highly toxic to non-fouling marine species at low ng/L concentrations, resulting in its banning in 2008 by the International Maritime Organization (Antizar-Ladislao 2008). As a result, there is high demand for a new generation of antifouling (AF) technologies to counteract biofouling. One such area is the development of antifoulants that exert their activity in a selective and non-toxic manner; that is, they deter organisms from settling rather than killing them.

In Nature, numerous strategies have been developed by a diverse range of organisms to counteract the risk of being colonized or overgrown. Sessile marine organisms often employ various physical and/or chemical strategies to mitigate the threat of fouling epibionts and predators (Proksch 1994; Proksch et al. 2010). Sponges, for example, and their symbiotic bacteria are capable of producing a large variety of complex metabolites, and it is suspected that some of these natural products are produced to repel settling species. Accordingly, marine natural products represent a particularly valuable resource in the search for new AF compounds (Qian et al. 2015; Fusetani 2011). In addition to producing an arsenal of AF compounds, marine and terrestrial plants also use allelopathic phytochemicals to suppress competitive species. These allelochemicals have the ability to prevent the establishment of competitive plant species and represent important defensive chemical agents for many plants and algae (Nilsson and Wardle 2005).

**Fig. 1:**
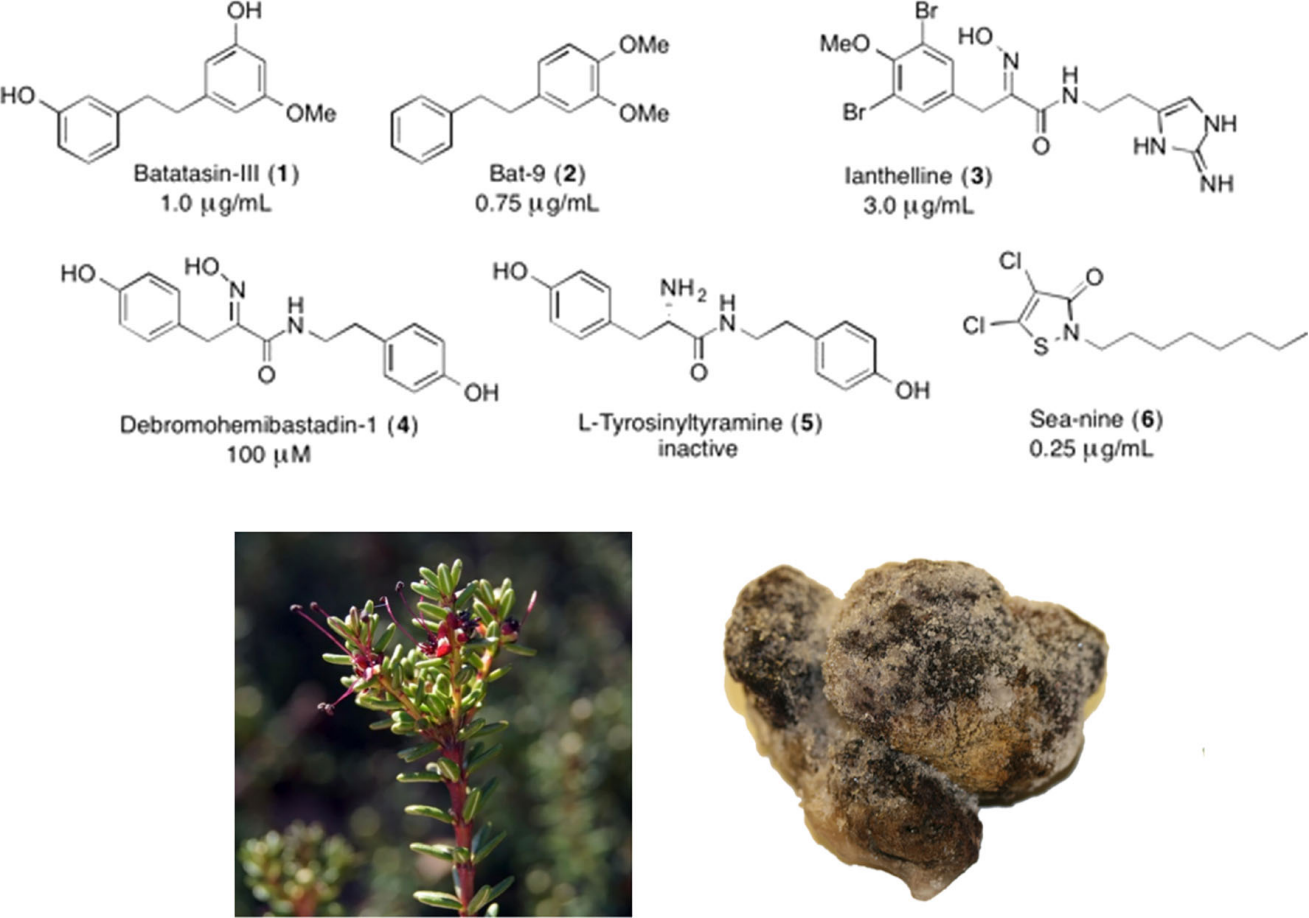
Top: representative antifouling compounds and corresponding IC_50_ activities against *Balanus improvises* larvae settlement; **1** (Moodie et al. 2017b), **2** (Moodie et al. 2017b), **3** (Hanssen et al. 2014), **4** (minimum significant dose to inhibit settlement) (Ortlepp et al. 2007), **5** (Ortlepp et al. 2007), **6** (Moodie et al. 2017b). Lower left panel: *Empetrum nigrum* (the common crowberry), a very prolific producer of **1** which is used to control competing plant species and recently shown to also be a highly potent marine antifoulant. Lower right panel: Specimen of the Arctic sponge *Stryphnus fortis* from which the oxime containing marine antifoulant ianthelline has been isolated

Intrigued whether the allelopathic activity of terrestrially derived natural products can be used to yield effective antifoulants in a marine setting, we recently investigated the AF activity of the allelopathic dihydrostilbene compound batatasin-III (**1**) (Fig. 1) (Moodie et al. 2017a, b). Batatasin-III is produced by a number of terrestrial plants, including the crowberry (*Empetrum nigrum*), where it accumulates in high amounts (up to 6% of the dry leaf weight). Upon leaching into the surrounding soil, it imparts an allelopathic effect, suppressing seedling growth and germination (González et al. 2015; Nilsson and Wardle 2005; Bråthen et al. 2010). During our studies, we established that compound **1**, and a number of the tested synthetic dihydrostilbene analogs (including Bat-9, **2**; Fig. 1) exhibited strong activity against marine microfouling and macrofouling species. Furthermore, several of the prepared compounds were shown to exert their AF effect by a non-toxic reversible mechanism.

Recent work from Takamura et al. (2017) describes an approach where the authors fused the structural motifs of the natural antifoulants butenolide and geraniol to generate a library of AF hybrid molecules. Given the known AF activity of these structural features, they rationalized that their combination could have a synergistic effect, providing AF entities with improved bioactivity. Combining different bioactive ligands/pharmacophores into a single molecule is a strategy currently employed in medical research where such multi-target-directed ligands (MTDLs) are investigated as improved drug leads, for example, in the treatment of neurodegenerative disorders (Rochais et al. 2015; Olsen et al. 2016). The recently published work by Takamura et al. represents the first attempt to extrapolate the MTDL strategy into a marine setting. Their resulting butenolide geraniol hybrid compounds were all found to inhibit the settlement of *Balanus amphitrite* cyprid larvae at lower concentrations (IC_50_ = 3–1.3 μg/mL) than the individual butenolide and geraniol components (Takamura et al. 2017).

**Fig. 2:**
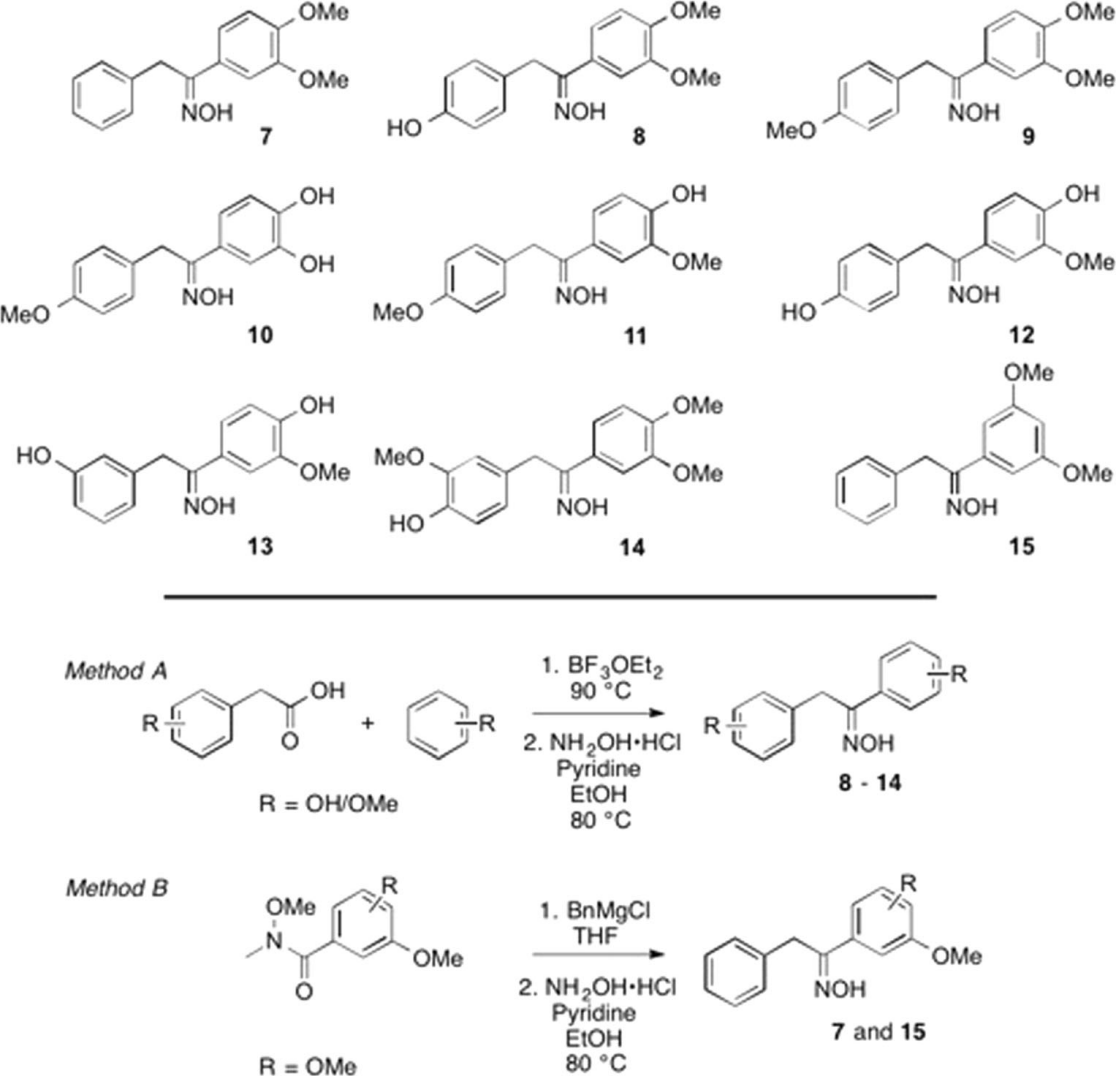
Hybrid dihydrostilbene-oxime compounds **7**–**15** and two general synthetic routes employed

A considerable number of effective natural marine antifoulants, for example, ianthelline (**3**), psammaplin A, and debromohemibastadin-1 (**4**), contain the oxime functionality (Hanssen et al. 2014; Ortlepp et al. 2007; Le Norcy et al. 2017a, b) in a homobenzylic position. The planar oxime provides structural rigidity to the molecules, decreasing rotational freedom, and studies by Proksch and coworkers have established the crucial role of the oxime for the AF activity of the bastadin family of compounds (Bayer et al. 2011; Ortlepp et al. 2007). In analogy to the recently reported AF hybrid strategy, we decided to investigate whether hybrid dihydrostilbene-oxime compounds would yield effective AF agents. Compound **2** was chosen as a lead structure given its ng/mL activity against key strains of microalgae and marine bacteria involved in biofilm formation, and its low μg/mL activity against *Balanus improvisus* and ascidian *Ciona savignyi* settlement inhibition (IC_50_, 0.75 and 1.1 μg/mL, respectively). Compound **2** also displayed low toxicity against the latter two fouling species and, in particular, effectively inhibited the settlement of *C. savignyi* even after 5 days (Moodie et al. 2017b). A library of compounds based on lead compound **2** was rationally designed and synthesized, containing the 3,4-dimethoxy-substitution pattern found in **2**, and variants thereof. Dihydrostilbene-oxime hybrids with further functionalized phenyl rings were also synthesized (compounds **7**–**15**; Fig. 2).

To try and encompass a range of species representative of the fouling process, the effect of the library on the adhesion and growth of ten marine bacterial and four microalgal species is described. In addition, the effect of these compounds on the settlement of barnacle *Balanus improvisus* larvae was also investigated to provide insight in their inhibitory effect on a major macrofouler. Comparisons are made with both reported natural antifoulants containing relevant structural features, and with the commercial antifoulants Sea-nine^™^ which was employed as a positive control.

## Materials and Methods

### Chemical Synthesis

A library of nine dibenzylic hybrid molecules based on both the 3,4-dimethoxy substituents, found in AF compound **2**, and the oxime motif were designed. Compounds **8**–**14** were prepared via boron trifluoride diethyl etherate catalyzed Friedel-Crafts acylation reactions between appropriately substituted phenyl acetic acids and benzenes (Xiao et al. 2007) followed by oxime formation (method A). Compounds **7** and **15** were synthesized by addition of benzyl magnesium chloride to a suitably functionalized Weinreb amide, and subsequent oxime formation (method B). The oximes were obtained as single isomers, of which the geometry was not determined. General experimental procedures and compound characterization are provided in the supplementary material.

Representative example of oxime synthesis using method A.

#### 1-(3,4-Dihydroxyphenyl)-1-Hydroxyimino-2-(4′-Methoxyphenyl)-Ethane (**10**)

Catechol (60 mg, 0.5 mmol) and 4-methoxyphenyl acetic acid (90 mg, 0.5 mmol) were dissolved in BF_3_•OEt_2_ (3 mL). The reaction was heated at 90 °C for 3 h, cooled to room temperature, quenched with aqueous sodium acetate (5 mL, wt 10%), and extracted into ethyl acetate (3 × 10 mL). The combined organic extracts were washed with brine and dried over Na_2_SO_4_. The solvent was removed under reduced pressure and the resulting residue purified by column chromatography (petroleum ether–ethyl acetate) to afford the desired deoxybenzoin (Ng et al. 2009) (70 mg, 51%) as an amorphous solid. Hydroxylamine hydrochloride (19 mg, 0.27 mmol) and pyridine (19 μL, 0.23 mmol) were added to a solution of deoxybenzoin (20 mg, 0.08 mmol) in absolute ethanol (3 mL). The reaction was heated at 80 °C for 3 h before cooling to ambient temperature. The solvent was removed in vacuo, and the residue was dissolved in ethyl acetate, washed with water, brine, and dried over Na_2_SO_4_. Removal of solvent under reduced pressure afforded oxime **10** (14 mg, 67%) as an amorphous solid. IR (neat) ν_max_ 3495, 3293, 1610, 1510, 1432, 1304, 1276, 1232, 1179, 1022, 959, 865, 756 cm^−1^; ^1^H NMR (CD_3_OD, 400 MHz) δ 7.14 (2H, d, *J* = 8.6 Hz), 7.09 (1H, d, *J* = 2.1 Hz), 6.95 (1H, dd, *J* = 8.3, 2.1 Hz), 6.77 (2H, d, *J* = 8.7 Hz), 6.69 (1H, d, *J* = 8.3 Hz), 4.03 (2H, s), 3.72 (3H, s); ^13^C NMR (CD_3_OD, 101 MHz) δ 159.5, 158.3, 147.5, 146.1, 130.7, 130.7, 129.4, 119.8, 115.9, 114.8, 114.7, 55.6, 31.7; HRMS *m*/*z* 274.1075 (calcd for C_15_H_16_NO_4_: 274.1074).

Representative example of oxime synthesis using method B.

#### 1-(3,5-Dimethoxyphenyl)-1-Hydroxyimino-2-Phenylethane (**15**)

A solution of *N*,3,5-trimethoxy-*N*-methyl benzamide (Romines et al. 2006) (74 mg, 0.3 mmol) in THF (3 mL) under an argon atmosphere was cooled to 0 °C and treated with benzyl magnesium chloride (328 μL, 0.6 mmol, 2.0 M in THF). The reaction was allowed to warm to room temperature and stirred for 12 h. After quenching with saturated NH_4_Cl solution, the reaction mixture was extracted with ethyl acetate (2 × 10 mL). The combined organic extracts were washed with water, brine, and dried over Na_2_SO_4_. The solvent was removed in vacuo, and the resulting reside was purified by column chromatography (petroleum ether:ethyl acetate) to afford the corresponding deoxybenzoin (Ikeda et al. 1977) (71 mg, 85%). The methodology for oxime formation described in method A furnished **15** (Ikeda et al. 1977) (28 mg, 74%, 0.1 mmol scale). IR (neat) *ν*_max_ 3415, 1587, 1348, 1200, 1162, 966, 946, 843, 818, 703 cm^−1^; ^1^H NMR (CDCl_3_, 400 MHz) δ 7.26–7.24 (4H, m), 7.21–7.14 (1H, m), 6.78 (2H, d, *J* = 2.3 Hz), 6.45 (1H, t, *J* = 2.3 Hz), 4.17 (2H, s), 3.74 (6H, s); ^13^C NMR (CDCl_3_, 101 MHz) δ 160.7, 157.5, 137.5, 136.5, 128.6, 128.6, 126.4, 104.8, 101.5, 55.4, 32.3; HRMS *m*/*z* 294.1106 (calcd for C_16_H_17_NNaO_3_: 294.1101).

### Marine Organisms

Cyprid larvae of *B. improvisus* were reared in a laboratory cultivating system at Tjärnö Marine Biological Laboratory, University of Gothenburg, Sweden, as previously described by Berntsson et al. (2000). Four pure, but non-axenic, marine microalgae (obtained from Algobank, Caen, France) and 10 marine bacterial strains were used (Table 1) as representative microfoulers. These strains represent fouling species encountered in both estuarine and marine environments (Moodie et al. 2017b). The bacteria were grown at 26 °C in a marine medium, composed of 0.5% peptone (neutralized bacteriological peptone, Oxoid Ltd.) in filtered (Whatman 1001–270, pore size 11 μm) natural seawater. Microalgae were grown and maintained at 22 °C in F/2 medium.

**Table 1:** Biofouling microorganisms included in present study

Species	Abbreviation	Code
Microalgae		Algobank code
*Halamphora coffeaeformis*		AC 713
*Pleurochrysis roscoffensis*		AC 32
*Cylindrotheca closterium*		AC 170
*Porphyridium purpureum*		AC 122
Marine bacteria		ATCC^a^
*Vibrio aestuarianus*	*V.a.*	35,048
*Vibrio carchariae*	*V.c.*	35,084
*Vibrio harveyi*	*V.h.*	700,106
*Vibrio natriegens*	*V.n.*	14,058
*Vibrio proteolyticus*	*V.p.*	53,559
*Halomonas aquamarina*	*H.a.*	14,400
*Roseobacter litoralis*	*R.l.*	49,566
*Shewanella putrefaciens*	*S.p.*	8,071
*Pseudoalteromonas elyakovii*	*P.e.*	700,159
*Polaribacter irgensii*	*P.i.*	700,398

^a^American tissue culture code

### Antibacterial Assays

Bacterial strain adhesion and growth were determined according to the methods of Thabard et al. (2011). Bacterial suspensions (100-μL aliquots, 2 × 10^8^ colony forming units/mL) were aseptically added to the compound containing microplate wells (10–0.01 μg/mL), and the plates were incubated for 48 h at 26 °C. Media only was used as a blank. Bacterial growth was monitored spectroscopically at 630 nm. The minimal inhibitory concentration (MIC) for bacterial growth was defined as the lowest concentration which results in a decrease in OD. After 48-h incubation time, the bacterial adhesion assay was conducted by emptying the wells and rinsing with sterile seawater (100 μL) to remove non-attached cells, and air-drying at room temperature. The residual bacterial biofilm was stained with aqueous crystal violet (100 μL, 0.3% *v*/*v*) and the OD measured at 595 nm (Sonak and Bhosle 1995). The MIC was defined as the lowest concentration of compound that, after 48-h incubation, produced a decrease of the OD at 595 nm. If inhibition was observed, toxicity tests were conducted. The well contents were transferred into a flask of fresh media, and growth was measured after 5 days of additional incubation. The mode of action was deemed biostatic if an increase in OD was measured at 595 nm (Moodie et al. 2017a).

### Antimicroalgal Assays

Microplates containing the compounds in ranging concentrations (10–0.01 μg/mL) were prepared from MeOH stock solutions as previously described (Trepos et al. 2014; Moodie et al. 2017a). Microalgal stock solutions were prepared using the chlorophyll analysis methodology of Chambers et al. (2011). The pretreated microplate wells were treated with 100 μL of the algal stock solutions (0.1 mg chlorophyll *a*/mL). The plates were then incubated for 5 days at 20 °C under constant light exposure (140 μmol m^−2^ s^−1^). Both microalgal adhesion and growth inhibition were measured. Growth was determined by analysis of liberated chlorophyll *a* after centrifugation and methanol addition. Chlorophyll *a* was quantified fluorometrically. MIC value for algal growth was defined as the lowest concentration yielding a decrease in chlorophyll *a* content. Microalgal adhesion was determined in an analogous manner where the non-attached algal cells were removed prior to methanol addition (100 μL), releasing chlorophyll *a* from the remaining algal biofilms. The MIC for adhesion was defined as the lowest compound concentration causing a reduction in optical density. Toxicity experiments were performed in an analogous manner to those described for the bacterial assays.

### Balanide Settlement Inhibition

Stock solutions of compounds in DMSO were serially diluted in untreated polystyrene Petri dishes containing 10 mL of filtered (0.2 μm) seawater, affording final concentrations ranging from 0.1 to 10 μg/mL. Freshly molted balanide cyprids (18–22) were added to each Petri dish and incubated at ambient temperature (20–25 °C) for 5 days. Cyprid metamorphosis was assessed using a dissecting microscope where the numbers of juvenile settled, free-swimming, and dead cyprids were noted. Initial compound screening was conducted at 5 μg/mL, and full IC_50_ determination was performed only on compounds displaying > 50% inhibition at that concentration. The IC_50_ was defined as the concentration preventing 50% of the cyprid settlement on the Petri dish surface. Each concentration was replicated four times (*n* = 4), and dishes containing 0.1% of DMSO were used as negative controls. The commercial AF agent Sea-nine^™^ was employed as a positive control.

## Results and Discussion

Marine biofouling is a highly complex and dynamic phenomenon, which is influenced by a range of processes at the physical, chemical, and biological levels (Callow and Callow 2011). The initial adsorption of organic molecules to a surface instigates a rapid settlement of microfouling organisms (e.g., marine bacteria and microalgae). The resulting biofilm provides a substratum for the attachment of the macrofouling macroalgae and invertebrates, which require longer settlement times. Consequently, to evaluate the potential of AF compounds, it is useful to survey a range of species that are representative of the whole fouling process (Briand 2009). In the current study of compounds **7**–**15**, bioassays were conducted that cover a spectrum of marine fouling organisms, including bacteria and microalgae (10 and four species, respectively) and the macrofouling barnacle, *Balanus improvisus*. As a relevant positive control, data for the commercial AF booster Sea-nine^™^ is included. Comparisons are also made with other relevant natural AF compounds.

Given the ability of microfouling epibionts to potentially encourage settlement of the more physically imposing macrofouling species (Qian et al. 2007), primary studies focused on the inhibition of both the adhesion and growth of marine bacteria and microalgal species. Even a thin slimy microfouling layer can induce a significant increase in drag for a vessel (Molino and Wetherbee 2008). To remain within a concentration regime of relevance for commercial AF applications, only compounds demonstrating minimum inhibitory concentrations of 10 μg/mL or below were considered active in the current study (Rittschof 2001; Trepos et al. 2014). Four of the nine compounds, **9**, **13**, **14**, and **15**, displayed inhibitory activities against bacterial adhesion at these low concentrations. Only compounds **14** and **15** displayed significant inhibitory effects against bacterial adhesion (Table 2). Of the five bacterial strains that were sensitive to the screened compounds, four of them were of the vibrio genus. Compound **13** represented the most potent compound against a single species with an MIC of 0.1 μg/mL against *V. aestuarianus*. This bacterium is of significant interest to the aquaculture industry as it has been linked to massive mortalities of the commercially important pacific oyster *Crassostrea gigas* (Barbosa Solomieu et al. 2015). In comparison, ianthelline was, in general, not effective at inhibiting bacterial adhesion (Hanssen et al. 2014).

**Table 2:** MIC (μg/mL) of compounds **9**, **13**, **14**, and **15** against the adhesion of marine bacteria

Compound^a^	*V.a.*	*V.c.*	*V.h.*	*V.p.*	*H.a.*
**9**	–^b^	–	–	–	10
**13**	0.1	–	–	–	–
**14**	10	10	10	–	10
**15**	10	10	10	–	10
Sea-nine^™c^	1	< 0.01	1	0.01	< 0.01
Ianthelline^d^	0.1	–	–	–	–
**2** ^e^	–	–	–	–	–

Tested strains: *Vibrio aestuarianus*, *Vibrio carchariae*, *Vibrio harveyi*, *Vibrio natriegens*, *Vibrio proteolyticus*, *Halomonas aquamarina*, *Roseobacter litoralis*, *Shewanella putrefaciens*, *Polaribacter irgensii*, and *Pseudoalteromonas elyakovii.* No activity was observed for all compounds against *V.n*., *R.l*., *S.p*., *P.e*., and *P.i*

*MIC* minimum inhibitory concentration

^a^Compounds **7**, **8**, **10**, **11**, and **12** were inactive against the adhesion of all bacteria tested up to 10 μg/mL

^b^Not active at > 10 μg/mL

^c^Data from Trepos et al. (2015)

^d^Data from Hanssen et al. (2014)

^e^Data from Moodie et al. (2017b)

In terms of bacterial growth inhibition, a greater breadth of activity was observed as shown in Table 3, where *P. irgensii* was the only bacterial species that was resilient to any members of the compound library at 10 μg/mL.

**Table 3:** MIC (μg/mL) of compounds **7**–**14** against the growth of marine bacteria

Compound^a^	*V.a.*	*V.c.*	*V.h.*	*V.n.*	*V.p.*	*H.a.*	*R.l.*	*S.p.*	*P.e.*
**7**	10	10	1	–^b^	–	–	–	–	–
**8**	–	–	–	–	–	10	–	1	–
**9**	–	–	–	–	–	10	–	0.1	–
**10**	–	–	–	10	–	–	–	–	1
**11**	–	–	–	–	–	10	–	–	–
**12**	–	–	–	–	–	0.1	1	0.1	10
**13**	–	–	–	1	–	–	–	–	–
**14**	–	–	–	–	–	–	–	10	–
Sea-nine^™c^	< 0.01	< 0.01	1	1	0.01	0.1	1	1	0.1
Ianthelline^d^	0.1	–	10	–	10	–	1	0.1	1
**2** ^e^	10	–	–	–	–	0.01	10	0.1	10

Tested strains: *Vibrio aestuarianus*, *Vibrio carchariae*, *Vibrio harveyi*, *Vibrio natriegens*, *Vibrio proteolyticus*, *Halomonas aquamarina*, *Roseobacter litoralis*, *Shewanella putrefaciens*, *Polaribacter irgensii*, and *Pseudoalteromonas elyakovii*. No activity was observed for all compounds against *P.i*

*MIC* minimum inhibitory concentration

^a^Compound **15** was inactive against the growth of all bacteria tested up to 10 μg/mL

^b^Not active at > 10 μg/mL

^c^Data from Trepos et al. (2015)

^d^Data from Hanssen et al. (2014)

^e^Data from Moodie et al. (2017b)

With the exception of **15**, all compounds displayed activity against at least one bacterial strain. Compound **12** was active against four strains, in particular against *H. aquamarina* and *S. putrefaciens* (0.1 μg/mL for both), but was inactive in the adhesion assays, a similar antibacterial profile to the natural product ianthelline (Hanssen et al. 2014). The latter species is involved with microbial induced corrosion of steel surfaces, a problem of significance in the food processing industry and also for marine constructions (Bagge et al. 2001). In comparison, compounds **8**, **9**, **10**, **11,** and **13**, which are also tri-substituted, lack both the potency of **12** and its ability to effect more than two bacterial species, suggesting that the 4,4′-dihydroxy-3-methoxy motif may affect this bioactivity. Compound **7** showed modest activity that was restricted to bacteria of the *Vibrio* genus.

From the obtained bacterial data, it is clear that members of the dihydrostilbene-oxime hybrid library are capable of inhibiting bacterial adhesion and growth, but no general inhibitors were identified and the antibacterial activity was not pronounced. In comparison to other AF terrestrial natural products such as polygodial and batatasin-III (**1**), the hybrid compounds displayed a similar activity against the investigated marine bacteria (Moodie et al. 2017a, b). The inhibitory activity towards bacterial attachment was higher for selected compounds in comparison to the parent **2** but only towards four of the included bacterial strains. Toxicity testing of the compounds that displayed inhibitory behavior revealed that they did so in a bacteriostatic manner, suggesting a non-toxic mechanism.

In order to investigate a range of fouling organisms, a second class of microfoulers were included, microalgae. Microalgae contribute to the formation of slimy biofilms that increase both the weight and hydrodynamic drag of ocean going vessels, and therefore, compounds that abrogate this behavior are of commercial interest (Molino and Wetherbee 2008). Of the four microalgae studied, two diatom species were included (*H. coffeaformis* and *C. closterium*). *H. coffeaeformis* in particular is a commonly used model species for diatom adhesion and growth (Molino and Wetherbee 2008). Compounds **7**–**15** were screened for microalgal adhesion and growth, and the results are summarized in Table 4. With the exception of compounds **11** and **15**, the compounds exhibited strong inhibitory activity against the tested microalgae in terms of both adhesion and growth. *P. purpureum* displayed a lower sensitivity towards the analyzed compounds which correlates well with our previous studies indicating an ability to resist many natural antifoulants. Compounds **7** and **13** inhibited both the settlement and growth of all of the tested species and were particularly potent against *H. coffeaformis* and *P. roscoffensis*. Further substitution of **7** in the 4′ position and modification of the 3,4-phenolic functionality (compounds **8**–**12**) resulted in reduced antimicroalgal activities. These antialgal activities are high and superior to several reported natural antifoulants, including ianthelline, which displays poor antialgal activity (Qian et al. 2010, 2015; Fusetani 2011; Hanssen et al. 2014). However, no apparent beneficial effect arising from the inclusion of the oxime functionality is seen as several of the compounds display comparable antialgal activities as their parent dibenzyls (Moodie et al. 2017b). It is of note that **15**, the 3,5-dihydroxy isomer of **7**, was not significantly active against the microalgae either. After algal cells were transferred to fresh media and incubated for 5 days, normal growth resumed, which suggests that the active compounds operate via a non-toxic reversible mechanism. Given the known toxicity issues of commercial antifoulants, these results are interesting.

**Table 4:** MIC (μg/mL) of compounds **7**–**15** against the adhesion (*A*) and growth (*G*) of microalgae

Compound	*H. coffeaformis*		*P. roscoffensis*		*C. closterium*		*P. purpureum*	
	*A*	*G*	*A*	*G*	*A*	*G*	*A*	*G*
**7**	0.01	0.1	0.01	0.01	1	1	1	10
**8**	10	10	10	10	10	10	–^a^	–
**9**	10	10	10	–	10	10	–	–
**10**	1	1	0.1	1	10	10	–	–
**11**	–	–	–	10	–	10	–	10
**12**	10	10	10	10	–	–	–	–
**13**	0.01	0.1	1	0.1	1	1	10	1
**14**	10	10	10	10	10	10	–	–
**15**	10	–	–	–	–	–	–	–
Sea-nine^™b^	< 0.01	< 0.01	< 0.01	< 0.01	< 0.01	< 0.01	< 0.01	< 0.01
Ianthelline^c^	> 10	> 10	> 10	10	> 10	> 10	> 10	> 10
**2** ^d^	1	0.01	1	0.01	0.1	0.01	10	1

*MIC* minimum inhibitory concentration

^a^Not active at > 10 μg/mL

^b^Data from Trepos et al. (2015)

^c^Data from Hanssen et al. (2014)

^d^Data from Moodie et al. (2017b)

**Fig. 3:**
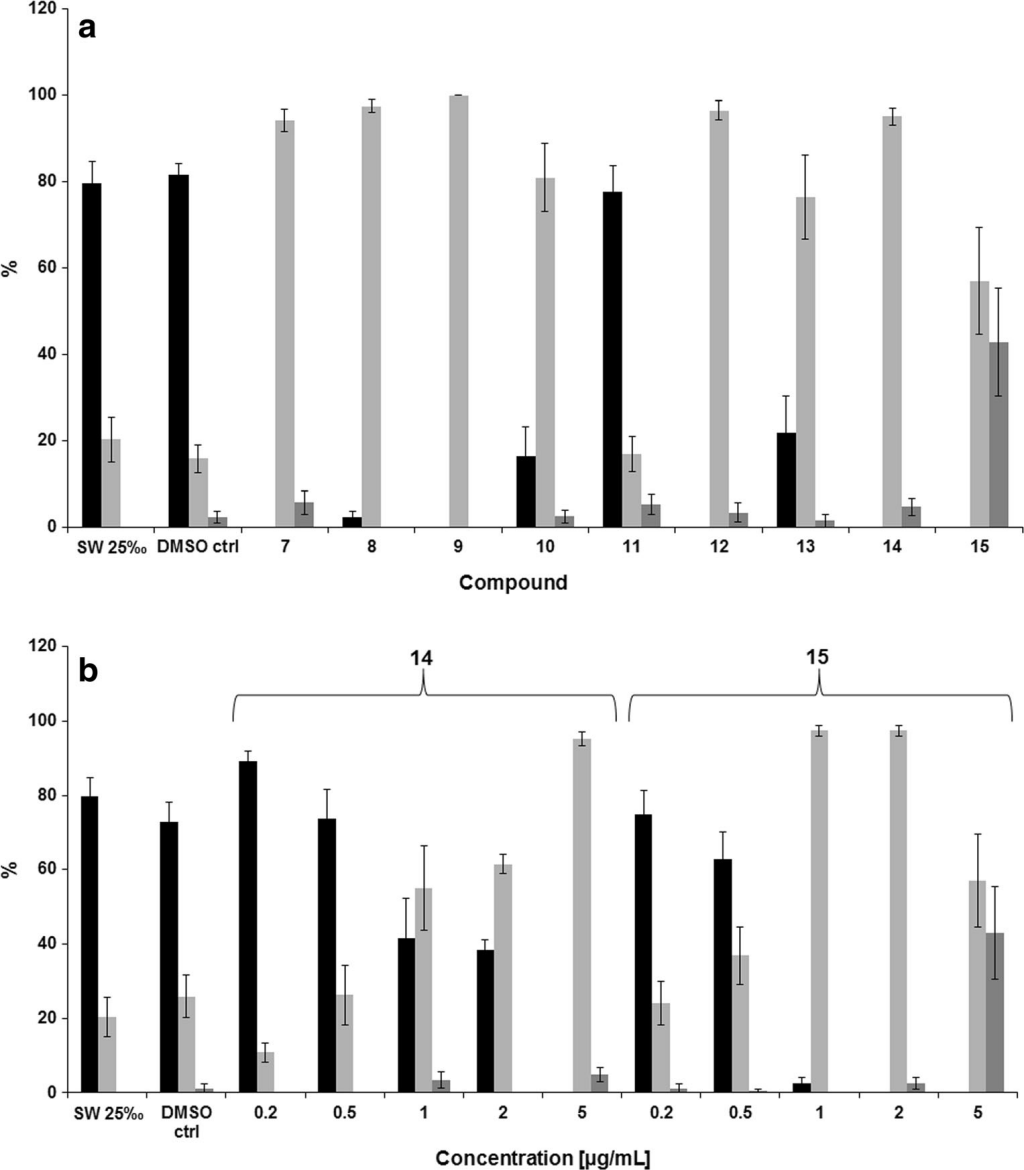
Effects of compounds **7**–**15** at 5 μg/mL on the settlement of *B. improvisus* cyprid larvae presented as percentages of settled (black columns), free swimming (light gray columns), and dead cyprids (dark gray columns) and given as means ± standard error (*n* = 4) (*A*). Filtered seawater (SW) and DMSO (0.1%, *v*/*v*) in SW were used as the negative control. Dose response analysis (0.2–5.0 μg/mL) of compounds **14** and **15** on the settlement inhibition of *B. improvisus* cyprid larvae (*B*). The columns are annotated as in method “A” above

While bacteria and microalgae are the primary settling biota during biofouling, the macrofouling species that follow them are often the more visible and physically daunting species. These can encompass soft fouling macroorganisms (i.e., seaweed, sponges, tunicates) and their harder calcareous counterparts (e.g., crustacea, mollusks, polychaete, tubeworms) (Qian et al. 2007). Barnacles are very common in fouling situations and represent a major biofouling organism at lower depths and in the splash zone. Barnacles are thus widely acknowledged as a useful model organism in AF research (Holm 2012). In the barnacle life cycle, the free-floating larval cyprids settle on a suitable surface before metamorphosis into their sessile form (Schumacher et al. 2007). Deterring cyprid settlement, and therefore mass colonization, in a non-toxic manner represents a significant challenge in AF research. In accordance, compounds **7**–**15** were investigated for their ability to inhibit the cyprid settlement and metamorphosis of the barnacle *Balanus improvisus.* Compounds were initially screened at a concentration of 5 μg/mL, and IC_50_ values for those deemed active were determined (Fig. 3 and Table 5). Additionally, toxicity was determined by considering the percentage of dead cyprids after incubation.

**Table 5:** Potency and toxicity of compounds **7**–**15** against the barnacle *B. improvisus*

Compound	IC_50_ (μg/mL)	Toxicity (%)^a^
**7**	2.5	5.8
**8**	5.0	0.0
**9**	1.5	0.0
**10**	5.0	2.6
**11**	> 5.0	5.3
**12**	2.5	3.5
**13**	5.0	1.6
**14**	1.0	4.8
**15**	0.75	42.9
Sea-nine^™^	0.25	n.d^b^
Ianthelline^c^	3.0	10.0
**2** ^d^	0.75	5.3

^a^Reported at 5 μg/mL. Toxicity for the negative control DMSO (0.1%, *v*/*v*) in filtered seawater was 2.4%

^b^Not determined

^c^Data from Hanssen et al. (2014)

^d^Data from Moodie et al. (2017b)

lThe tested library exerted a strong effect on balanide settlement inhibition with **11** the only inactive compound. The inactivity of compound **11** is surprising considering that all the other closely related compounds completely inhibited cyprid settlement at 5.0 μg/mL. The most potent inhibitor was the 3,5-dimethoxy substituted **15** (IC_50_ = 0.75 μg/mL), but this activity was accompanied by high toxicity. The remaining compounds yielded IC_50_ values that ranged between 1.0 and 5.0 μg/mL and, importantly, displayed very low toxicity at 5.0 μg/mL, comparable to that of the negative control DMSO (0.1%, *v*/*v*) in filtered seawater (Table 5).

Considering activity and toxicity, the tetra-substituted **14** was the best performing compound (IC_50_ 1.0 μg/mL, 4.8% cyprid mortality at 5.0 μg/mL). Although weaker than the positive control Sea-nine^™^, these inhibitory activities are higher than a large number of reported AF natural products and comparable to well-studied potent natural antifoulants such as barettin, ianthelline, polygodial, synoxazolidinone A & C, oroidin, butenolide, and geraniol (Fusetani 2011; Qian et al. 2015; Hanssen et al. 2014; Trepos et al. 2014). Compounds **14** and **15** displayed inhibitory properties in parity with the butenolide and geraniol hybrids recently reported by Takamura et al. (2017). Compound **7** was less active than its parent dihydrostilbene **2** (2.5 vs 0.75 μg/mL, respectively), advocating that, in this case, the addition of the oxime functionality did not provide improved activity. Examining the influence of structure (polarity, hydrogen bonding formation capacity) on settlement inhibition gave no clear relationships (data not included), suggesting that these compounds might exert their activity on multiple cellular targets.

A number of the tested dihydrostilbene-oxime hybrids were effective antifoulants, in particular against microalgae and balanide larval settlement, where the activities were comparable or better than many previously reported antifoulants (Fusetani 2011; Qian et al. 2015). The general activity against diatoms *H. coffeaformis* and *C. closterium* is promising as it has been shown that AF coatings can struggle to minimize the slime formation, which is influenced by these species (Molino and Wetherbee 2008). Overall, the compounds were less effective at adhesion, and growth inhibition of the tested marine bacteria strains, suggesting that these hybrid molecules are not effective over the full range of fouling organisms. However, it is of note that, compounds **11** and **15** aside, both high activity and very low toxicity was observed against barnacle larvae. Furthermore, against microalgae and marine bacteria, the inhibitory effects were reversible, suggesting a non-toxic mode of action(s).

As embodied by the hybrid approach of Takamura et al. (2017), the current project aimed to investigate if combining the dihydrostilbene scaffold with the oxime functional motif could access improved antifoulants. The majority of the tested compounds were indeed highly efficient antifoulants but they did not provide significant synergistic advantages over our previously reported batatasin library (Moodie et al. 2017b). The AF geraniol hybrids prepared by Takamura and coworkers displayed an increased activity compared to their parent compounds, not dictated by general physicochemical properties such as polarity and hydrogen bonding capacity (data not shown). The prepared hybrids are nevertheless twice as large, in terms of molecular weight, as the parent geraniol and the added molecular bulk may explain the increased activity of the hybrids. It is not known if the hybrids exert their AF activity by the modes of action of their parent compounds. The presently studied compounds are very similar in size to the parent dihydrostilbene compounds and also display similar AF properties, and hence, it is unclear if the hybrid approach represents a general method to produce improved antifoulants. A careful choice of combined molecular functionalities appears to be necessary to obtain significant improvements.

In particular, comparison can be made between **7** and **15**, and their non-oxime containing counterparts. Against balanide settlement inhibition, similar IC_50_ values were observed for the four compounds; however, the oxime motif of **15** significantly increased toxicity at 5 μg/mL in comparison to its dihydrostilbene analogue (42.9 vs 7.7%, respectively) (Moodie et al. 2017b). The oxime group of **15** diminished activity against the inhibition of both adhesion and growth of microalgae. It did however provide inhibitory activity against the adhesion of four species of marine bacteria. Both **7** and **2** were inactive against bacterial adhesion, but displayed growth inhibition against different species. Shifts in their respective inhibitory profiles against microalgal settlement and growth were also noted. While Proksch and co-workers noticed that the oxime motif was crucial for inhibiting *B. improvisus* larval settlement (i.e., **4** (100 μM) vs **5** (inactive); Fig. 1); in our previous studies, the bibenzyl scaffold alone still yielded effective AF compounds, suggesting a different mode of action to the bastadin-type compounds (Ortlepp et al. 2007). As a consequence of their biosynthesis from tyrosine, the oxime motifs of **3** and **4** and numerous other structurally similar marine natural products (Lindel and Hentschel 2009) are adjacent to an amide. Compound **4** was shown to inhibit blue mussel phenoloxidase, likely due to complexation of its α-oxo-oxime motif to the copper(II) ion containing catalytic center (Bayer et al. 2011). It could be also be speculated that this functionality would enable intramolecular hydrogen bonding interactions that influence bioactivity (Rappoport and Liebman 2009). Given the lack of an α-oxo group in the compounds of the current study, these modes of action may be not be applicable in our case. It is currently not known whether the oximes operate on similar cellular targets to the dihydrostilbenes, or if they induce a shift in the mode of action(s). The geometries of the oximes used in this study were not determined, and the potential influence that these orientations may have on bioactivity requires further investigation.

The bioactivity data obtained from compounds **7**–**15** against 15 different fouling organisms does not yield any general structure activity relationships (SAR). This is likely reflective of the breadth of studied species, and therefore of their diverse and evolutionary distinct cellular pathways. Whereas the potency of dihydrostilbenes against balanide cyprid inhibition could be linked to hydrophobicity, a similar link for the oxime hybrids was not noticed (Moodie et al. 2017b). A lack of narrow and clearly defined SARs in related bibenzyl compounds has been noted on several occasions (Moodie et al. 2017b; Hernandez-Romero et al. 2004; Oozeki et al. 2008; Trombetta et al. 2014). For example, Hernández-Romero et al. (2005) noticed that compounds functionalized with a mixture of methoxy- and phenol substituents improved herbicidal activity, but no trend was revealed for cytotoxicity in mammalian cell lines (Hernandez-Romero et al. 2005). Although structurally related dihydrostilbene-oxime compounds have been investigated in biological settings, including inhibition of NADH:ubiquinone oxidoreductase (Nicolaou et al. 2000) and catechol-*O*-methyl transferase (Learmonth et al. 2002), and urease in *Helicobacter pylori* (Li et al. 2009), the current study represents the first time that this scaffold has been employed in an AF capacity.

## Conclusion

Preventing marine biofouling using environmentally friendly technologies represents a significant challenge for the scientific and commercial sectors. Therefore, the development of small molecules that exert AF activities via non-toxic mechanisms is of importance. In the present study, we combine the dihydrostilbene and oxime structural motifs, which have both independently shown inhibitory behaviors against fouling organisms, to construct a library of hybrid molecules. In general, these compounds displayed strong inhibitory behavior against the settlement and growth of a panel of marine microalgae, including two species of diatoms. Although the effect against marine bacteria was less pronounced, these compounds operate by a non-toxic mode of action(s), which is particularly encouraging. A number of the compounds were also effective at inhibiting the settlement of balanide larvae at low concentrations, which demonstrated their potency against a macrofouling species.

## Electronic Supplementary Material

ESM 1 (DOCX 1.47 mb)
