# Supplementary material for: Design and Biological Evaluation of Antifouling Dihydrostilbene Oxime Hybrids
Source: Mar Biotechnol (NY). 2018 Mar 13;20(2):257–67. doi: 10.1007/s10126-018-9802-z (PMC5889410; doi:10.1007/s10126-018-9802-z)
Supplement: Supplementary file 1 — (DOCX 1.47 mb) [file 10126_2018_9802_MOESM1_ESM.docx]

**Design and Biological Evaluation of Antifouling Dihydrostilbene Oxime Hybrids**

*Lindon W. K. Moodie,^1,2,*^ Gunnar Cervin,^3^ Rozenn Trepos,^4^ Christophe Labriere,^1^ Claire Hellio,^4^ Henrik Pavia,^3^ Johan Svenson^1,5^*

^1^ Department of Chemistry, UiT The Arctic University of Norway, Breivika N-9037, Tromsø, Norway

^2^ Current address, Department of Chemistry, Umeå University, SE-901 87, Umeå, Sweden

^3^ Department of Marine Sciences - Tjärnö, University of Gothenburg, SE-452 96 Strömstad, Sweden

^4^ Université de Bretagne Occidentale, Biodimar/LEMAR UMR 6539, Rue Dumont d’Urville, 29280 Plouzané, France

^5^ Current address, Department of Chemistry, Material and Surfaces, RISE Research Institutes of Sweden, Box 857, SE-501 15 Borås, Sweden

*Corresponding author: Lindon W. K. Moodie; E-mail: [Lindon.moodie@umu.se](mailto:Lindon.moodie@umu.se)

(ORCID 0000-0002-9500-4535)

Supporting Information Contents

**General Experimental Procedures 3**

**Synthesis of compounds 7-15 3 - 5**

**Figure S1.** ^1^H-NMR of **7** in CD_3_OD **7**

**Figure S2.** ^13^C-NMR of **7** in CD_3_OD **7**

**Figure S3.** ^1^H-NMR of **8** in CD_3_OD **8**

**Figure S4.** ^13^C-NMR of **8** in CD_3_OD **8**

**Figure S5.** ^1^H-NMR of **9** in CD_3_OD **9**

**Figure S6.** ^13^C-NMR of **9** in CD_3_OD **9**

**Figure S7.** ^1^H-NMR of **10** in CD_3_OD **10**

**Figure S8.** ^13^C-NMR of **10** in CD_3_OD **10**

**Figure S9.** ^1^H-NMR of **11** in CD_3_OD **11**

**Figure S10.** ^13^C-NMR of **11** in CD_3_OD **11**

**Figure S11.** ^1^H-NMR of **12** in CD_3_OD **12**

**Figure S12.** ^13^C-NMR of **12** in CD_3_OD **12**

**Figure S13.** ^1^H-NMR of **13** in CD_3_OD **13**

**Figure S14.** ^13^C-NMR of **13** in CD_3_OD **13**

**Figure S15.** ^1^H-NMR of **14** in CD_3_OD **14**

**Figure S16.** ^13^C-NMR of **14** in CD_3_OD **14**

**Figure S17.** ^1^H-NMR of **15** in CDCl_3_ **15**

**Figure S18.** ^13^C-NMR of **15** in CDCl_3_ **15**

**References 16**

**General Experimental Procedures**

^1^H and ^13^C-NMR spectra were acquired on a Varian 7000e 400 MHz spectrometer. ^1^H Chemical shifts are reported in δ values relative to tetramethylsilane and referenced to the residual solvent peak (CD_3_OD: δ_H_ = 3.310, δ_C_ = 49.00 ppm; CDCl_3_: δ_H_ = 7.26 ppm, δ_C_ = 77.16 ppm). Coupling constants are reported in Hz. High-resolution mass spectroscopy was recorded on an LTQ Orbitrap XL Hybrid Fourier transform mass spectrometer from Thermo Scientific. Infrared spectra were recorded on an Avatar 320 FT-IR spectrometer from Nicolet. Solvents and reagents were purchased from commercial suppliers and used without further purification. Air-sensitive reactions were carried out under an argon atmosphere. Thin-layer chromatography was carried out on aluminum-backed plates coated with silica gel and visualized under UV light at 254 nm and ethanolic vanillin dip. Chromatography was carried out on silica gel using petroleum ether and ethyl acetate as eluents. All the compounds were tested at the purity shown in the SI. Names for novel compounds are depicted in italics. Spectral data for reported compounds is included if previously reported experimental data is incomplete. 3,4,*N*-Trimethoxy-*N*-methyl-benzamide (Yamazaki et al., 2012) and *N*,3,5-trimethoxy-*N*-methyl benzamide (Romines et al., 2006) were prepared accordingly to a reported protocol.

**Synthesis of compounds 7-15**

Compounds synthesised using Method A (see main text)

*1-(3,4-dimethoxyphenyl)-1-hydroxyimino-2-(4’-hydroxyphenyl)-ethane* (**8**)

Acylation (Medarde et al., 1994): 67% yield (0.7 mmol scale). Oxime formation: 93% yield (0.1 mmol scale). IR (neat) *ν*_max_ 3427, 1601, 1511, 1252, 1225, 1021, 964 cm^-1^; ^1^H NMR (CD_3_OD, 400 MHz) δ 7.26 (1H, d, *J* = 2.1 Hz), 7.15 (1H, dd, *J* = 8.4, 2.1 Hz), 7.06 (2H, d, *J* = 8.4 Hz), 6.87 (1H, d, *J* = 8.4 Hz), 6.65 (2H, d, *J* = 8.5 Hz), 4.06 (2H, s), 3.81 (3H, s), 3.78 (3H, s); ^13^C NMR (CD_3_OD, 101 MHz) δ 158.0, 156.7, 151.2, 150.1, 130.7, 130.4, 129.5, 121.0, 116.2, 112.2, 110.9, 56.3, 56.3, 31.6; HRMS *m*/*z* 310.1055 (calcd for C_16_H_17_NNaO_4_: 310.1050).

*1-(3,4-dimethoxyphenyl)-1-hydroxyimino-2-(4’-methoxyphenyl)-ethane* (**9**)

Acylation (Napolitano et al., 1983): 63% yield (0.6 mmol scale): IR (neat) *ν*_max_ 1672, 1513, 1417, 1242, 1149, 816 cm^-1^ ^1^H NMR (CDCl_3_, 400 MHz) δ 7.65 (1H, dd, *J* = 8.4, 2.0 Hz), 7.55 (1H, d, *J* = 2.0 Hz), 7.23 – 7.16 (2H, m), 6.89 – 6.79 (3H, m), 4.18 (2H, s), 3.93 (3H, s), 3.91 (3H, s), 3.78 (3H, s); ^13^C NMR (CDCl_3_, 101 MHz) δ 196.8, 158.6, 153.4, 149.2, 130.4, 129.9, 127.1, 123.5, 114.2, 110.8, 110.1, 56.2, 56.1, 55.4, 44.4; HRMS *m*/*z* 309.1101 (calcd for C_17_H_18_NaO_4_: 309.1097). Oxime formation: 69% yield (0.1 mmol scale). IR (neat) *ν*_max_ 3443, 2549, 1693, 1510, 1243, 1178, 1023, 817, 764 cm^-1^; ^1^H NMR (CD_3_OD, 400 MHz) δ 7.27 (1H, d, *J* = 1.9 Hz), 7.15 (2H, d, *J* = 8.7 Hz), 7.13 (1H, dd, *J* = 8.4, 2.1 Hz), 6.87 – 6.82 (1H, m), 6.76 (2H, d, *J* = 8.7 Hz), 4.08 (2H, s), 3.78 (3H, s), 3.76 (3H, s), 3.70 (3H, s); ^13^C NMR (CD_3_OD, 101 MHz) δ 159.5, 157.8, 151.2, 150.1, 131.3, 130.7, 130.4, 120.9, 114.8, 112.2, 110.9, 56.3, 56.3, 55.6, 31.5; HRMS *m*/*z* 324.1211 (calcd for C_17_H_19_NNaO_4_: 324.1206).

*1-(3-methoxy-4-hydroxyphenyl)-1-hydroxyimino-2-(4’-methoxyphenyl)-ethane* (**11**)

Acylation: 46% yield (0.6 mmol scale): ^1^H NMR (CDCl_3_, 400 MHz) δ 7.62 (dd, *J* = 8.3, 1.9 Hz, 1H), 7.56 (d, *J* = 2.0 Hz, 1H), 7.21 – 7.16 (m, 2H), 6.93 (d, *J* = 8.3 Hz, 1H), 6.89 – 6.83 (m, 2H), 4.17 (s, 2H), 3.91 (s, 3H), 3.78 (s, 3H); ^13^C NMR (CD_3_OD, 101 MHz) δ 196.8, 158.6, 150.5, 146.8, 130.4, 129.6, 127.1, 124.2, 114.2, 114.0, 110.5, 56.1, 55.4, 44.3. Oxime formation: 83% yield (0.1 mmol scale). IR (neat) *ν*_max_ 3384, 1509, 1244, 1224, 1028 cm^-1^; ^1^H NMR (CD_3_OD, 400 MHz) δ 7.23 (1H, d, *J* = 2.0 Hz), 7.15 (2H, d, *J* = 8.6 Hz), 7.05 (1H, dd, *J* = 8.3, 2.0 Hz), 6.77 (2H, d, *J* = 8.7 Hz), 6.72 (1H, d, *J* = 8.3 Hz), 4.07 (2H, s), 3.79 (3H, s), 3.71 (3H, s); ^13^C NMR (CD_3_OD, 101 MHz) δ 159.5, 158.2, 148.7, 148.7, 131.3, 130.7, 129.2, 121.1, 115.8, 114.8, 110.8, 56.3, 55.6, 31.6; HRMS *m*/*z* 310.1054 (calcd for C_16_H_17_NNaO_4_: 310.1050).

*1-(3-methoxy-4-hydroxyphenyl)-1-hydroxyimino-2-(4’-hydroxyphenyl)-ethane* (**12**)

Acylation (Ley et al., 2012): 48% yield (0.7 mmol scale). Oxime formation: 74% yield (0.1 mmol scale). IR (neat) *ν*_max_ 3327, 1596, 1512, 1261, 1220, 1173, 1028 cm^-1^; ^1^H NMR (CD_3_OD, 400 MHz) δ 7.22 (1H, d, *J* = 1.9 Hz), 7.06 (2H, d, *J* = 8.6 Hz), 7.05 (1H, dd, *J* = 8.3, 2.1 Hz), 6.72 (1H, d, *J* = 8.3 Hz), 6.65 (2H, d, *J* = 8.6 Hz), 4.04 (2H, s), 3.80 (3H, s); ^13^C NMR (CD_3_OD, 101 MHz) δ 158.4, 156.6, 148.7, 148.6, 130.7, 129.6, 129.3, 121.1, 116.2, 115.8, 110.9, 56.3, 31.7; HRMS *m*/*z* 296.0898 (calcd for C_15_H_15_NNaO_4_: 296.0893).

*1-(3-methoxy-4-hydroxyphenyl)-1-hydroxyimino-2-(3’-hydroxyphenyl)-ethane* (**13**)

Acylation: 66% yield (0.7 mmol scale): IR (neat) *ν*_max_ 3392, 3200, 1577, 1454, 1274, 1223, 1126, 692 cm^-1^; ^1^H NMR (CD_3_OD, 400 MHz) δ 7.63 (1H, dd, *J* = 8.3, 2.0 Hz), 7.55 (1H, d, *J* = 1.9 Hz), 7.10 (1H, t, *J* = 7.8 Hz), 6.85 (1H, d, *J* = 8.3 Hz), 6.77 – 6.68 (2H, m), 6.64 (1H, dd, *J* = 8.2, 2.1 Hz), 4.17 (2H, s), 3.87 (3H, s); ^13^C NMR (CD_3_OD, 101 MHz) δ 199.0, 158.6, 153.4, 149.0, 138.2, 130.6, 129.9, 125.4, 121.6, 117.2, 115.8, 114.6, 112.5, 56.4, 45.9; HRMS *m*/*z* 281.0788 (calcd for C_15_H_14_NaO_4_: 281.0784). Oxime formation: 74% yield (0.1 mmol scale). IR (neat) *ν*_max_ 3350, 1587, 1516, 1259, 1220, 1028, 965, 741 cm^-1^; ^1^H NMR (CD_3_OD, 400 MHz) δ 7.24 (1H, d, *J* = 2.0 Hz), 7.05 (1H, dd, *J* = 8.3, 2.1 Hz), 7.04 (1H, t, *J* = 7.9 Hz), 6.75 – 6.67 (3H, m), 6.57 (1H, dd, *J* = 7.9, 2.0 Hz), 4.08 (2H, s), 3.81 (3H, s); ^13^C NMR (CD_3_OD, 101 MHz) δ 158.5, 157.8, 148.7, 148.7, 140.4, 130.3, 129.3, 121.1*, 116.6, 115.8, 114.0, 110.8, 56.3, 32.5 *Corresponds to two carbon signals; HRMS *m*/*z* 274.1078 (calcd for C_15_H_16_NO_4_: 274.1074).

*1-(3,4-dimethoxyphenyl)-1-hydroxyimino-2-(3’-methoxy-4’-hydroxyphenyl)-ethane* (**14**)

Acylation (Barclay et al., 1994): 79% yield (0.5 mmol scale). Oxime formation: 84% yield (0.1 mmol scale). IR (neat) *ν*_max_ 3293, 1602, 1513, 1271, 1250, 1224, 1146, 1020 cm^-1^; ^1^H NMR (CD_3_OD, 400 MHz) δ 7.27 (d, *J* = 2.0 Hz, 1H), 7.16 (dd, *J* = 8.4, 2.1 Hz, 1H), 6.87 – 6.82 (m, 2H), 6.68 – 6.63 (m, 2H), 4.07 (s, 2H), 3.79 (s, 3H), 3.77 (s, 3H), 3.75 (s, 3H); ^13^C NMR (CD_3_OD, 101 MHz) δ 158.0, 151.2, 150.1, 149.1, 146.3, 130.5, 129.8, 122.3, 121.0, 116.3, 113.4, 112.2, 111.0, 56.3*, 56.3, 32.0 *Corresponds to two carbon signals; HRMS *m*/*z* 340.1158 (calcd for C_17_H_19_NNaO_5_: 340.1155).

Compounds synthesised using Method B (see main text)

1-(3,4-dimethoxyphenyl)-1-hydroxyimino-2-phenylethane (**7**)

Grignard (Kaito et al., 2006) 74% yield (0.3 mmol scale). Oxime formation:(Chen et al., 2016) 70% yield (0.2 mmol scale). IR (neat) *ν*_max_ 3446, 3221, 1601, 1514, 1252, 1227, 1023, 717 cm^-1^; ^1^H NMR (CD_3_OD, 400 MHz) δ 7.28 (1H, d, *J* = 2.1 Hz), 7.25 – 7.17 (4H, m), 7.15 – 7.10 (2H, m), 6.82 (1H, d, *J* = 8.4 Hz), 4.16 (2H, s,), 3.77 (3H, s), 3.75 (3H, s); ^13^C NMR (CD_3_OD, 101 MHz) δ 157.4, 151.2, 150.1, 138.8, 130.3, 129.7, 129.4, 127.1, 120.9, 112.2, 110.8, 56.3, 56.3, 32.3; HRMS *m*/*z* 294.1102 (calcd for C_16_H_17_NNaO_3_: 294.1101).

**Figure S1.** ^1^H-NMR of **7** in CD_3_OD.

**Figure S2.** ^13^C-NMR of **7** in CD_3_OD.

**Figure S3.** ^1^H-NMR of **8** in CD_3_OD.

**Figure S4.** ^13^C-NMR of **8** in CD_3_OD.

**Figure S5.** ^1^H-NMR of **9** in CD_3_OD.

**Figure S6.** ^13^C-NMR of **9** in CD_3_OD.

**Figure S7.** ^1^H-NMR of **10** in CD_3_OD.

**Figure S8.** ^13^C-NMR of **10** in CD_3_OD.

**Figure S9.** ^1^H-NMR of **11** in CD_3_OD.

**Figure S10.** ^13^C-NMR of **11** in CD_3_OD.

**Figure S11.** ^1^H-NMR of **12** in CD_3_OD.

**Figure S12.** ^13^C-NMR of **12** in CD_3_OD.

**Figure S13.** ^1^H-NMR of **13** in CD_3_OD.

**Figure S14.** ^13^C-NMR of **13** in CD_3_OD.

**Figure S15.** ^1^H-NMR of **14** in CD_3_OD.

**Figure S16.** ^13^C-NMR of **14** in CD_3_OD.

**Figure S17.** ^1^H-NMR of **15** in CDCl_3_.

**Figure S18.** ^13^C-NMR of **15** in CDCl_3_.

**References**

Barclay LRC, Cromwell GR, Hilborn JW (1994) Photochemistry of a model lignin compound. Spin trapping of primary products and properties of an oligomer. Can J Chem 72**:**35-41

Chen R, Qi J, Mao Z, Cui S (2016) Rh(iii)-catalyzed C-H activation/cyclization of oximes with alkenes for regioselective synthesis of isoquinolines. Org Biomol Chem 14**:**6201-6204

Kaito C, Sakamoto K, Sakamoto M, Yamauchi A, Kihara M (2006) Synthesis of new 1,2-diphenyl-4,5-dihydro-3H-3-benzazepines. Heterocycles 68**:**2319-2326

Ley JP, Dessoy M, Paetz S, Blings M, Hoffmann-Lücke P, Reichelt KV, Krammer GE, Pienkny S, Brandt W, Wessjohann L (2012) Identification of Enterodiol as a Masker for Caffeine Bitterness by Using a Pharmacophore Model Based on Structural Analogues of Homoeriodictyol. J Agric Food Chem 60**:**6303-6311

Medarde M, De Clairac, RP-L, López JL, Feliciano AS (1994) A Versatile Approach to the Synthesis of Combretastatins. J Nat Prod 57**:**1136-1144

Napolitano E, Giannone E, Fiaschi R, Marsili A (1983) Influence of alkoxyalkyl substituents in the regioselective lithiation of the benzene ring. J Org Chem 48**:**3653-3657

Romines KR, Freeman GA, Schaller LT, Cowan JR, Gonzales SS, Tidwell JH, Andrews CW, Stammers DK, Hazen RJ, Ferris RG, Short SA, Chan JH, Boone LR (2006) Structure−Activity Relationship Studies of Novel Benzophenones Leading to the Discovery of a Potent, Next Generation HIV Nonnucleoside Reverse Transcriptase Inhibitor. J Med Chem 49**:**727-739

Yamazaki Y, Sumikura M, Masuda Y, Hayashi Y, Yasui H, Kiso Y, Chinen T, Usui T, Yakushiji F, Potts B, Neuteboom S, Palladino M, Lloyd GK, Hayashi Y (2012) Synthesis and structure-activity relationships of benzophenone-bearing diketopiperazine-type anti-microtubule agents. Bioorg Med Chem 20**:**4279-4289
